# Distribution of Methylene Tetrahydrofolate Reductase Gene Polymorphisms in Women of Childbearing Age in Tai'an

**DOI:** 10.1155/ijog/8889232

**Published:** 2025-07-13

**Authors:** Bo Zhou, Chengzhen Gao, Lirong Chen, Yumei Wang, Shufang Zhang, Chao Hou, Xiangyang An

**Affiliations:** ^1^Postdoctoral Workstation, The Affiliated Tai'an City Central Hospital of Qingdao University, Tai'an, Shandong, China; ^2^School of Medical Equipment, Shenyang Pharmaceutical University, Shenyang, Liaoning, China; ^3^Pharmaceutical Companies, Lunan Pharmaceutical Group Co., Ltd., Linyi, Shandong, China; ^4^Department of Ultrasound, The Affiliated Tai'an City Central Hospital of Qingdao University, Tai'an, Shandong, China; ^5^Clinical Pharmacy, The Affiliated Tai'an City Central Hospital of Qingdao University, Tai'an, Shandong, China; ^6^Geriatric Neurology, The Affiliated Tai'an City Central Hospital of Qingdao University, Tai'an, Shandong, China; ^7^Maternal-Fetal Medicine Ward, The Affiliated Taian City Central Hospital of Qingdao University, Tai'an, Shandong, China

**Keywords:** gene polymorphism, methylenetetrahydrofolate reductase, women of childbearing age

## Abstract

**Objective:** The objective was to explore the distribution characteristics of methylenetetrahydrofolate reductase *MTHFR* 677C > T and 1298A > C gene polymorphisms among women of childbearing age in the Tai'an area.

**Method:** A total of 243 women of childbearing age, the age ranged from 20 to 46 years, with a mean age of 31.0 ± 5.1 years, attending the Eugenics Clinic of Tai'an City Central Hospital between January 2018 and April 2019 were selected. The *MTHFR* 677C > T and 1298A > C gene polymorphisms were determined by fluorescence in situ hybridization.

**Result:** Among 243 women of childbearing age, the *MTHFR* 677C > T genotype frequencies of CC, CT, and TT were 14.4%, 44.9%, and 40.7%, respectively, and the C and T Allele frequencies were 36.8% and 63.2%, respectively. The *MTHFR* 1298A > C genotype frequencies of AA, AC, and CC were 77.0%, 21.0%, and 2.0%, respectively. The A and C allele frequencies were 87.4% and 12.6%, respectively. The proportions of CC/AA, CC/AC, CC/CC, CT/AA, CT/AC, TT/AA, and TT/AC were 6.6%, 5.8%, 2.0%, 30.5%, 14.4%, 39.9%, and 0.8%. The proportion of women at moderate or severe risk of folic acid metabolism reached 87.6%. The *MTHFR* 677C > T gene mutation rate has obvious regional characteristics.

**Conclusion:** The *MTHFR* 677C > T and 1298A > C gene polymorphisms in Tai'an were obvious. About 87.6% of women of childbearing age were at medium or high risk of folic acid utilization.

## 1. Introduction

Folic acid is a water-soluble vitamin and an essential nutrient that cannot be synthesized in the body and must be obtained from external sources. It plays a crucial role in DNA synthesis and methylation [1]. The increased demands for folic acid during pregnancy, due to the rapid growth of the fetus, placenta, and maternal tissues, are approximately 5–10 times higher than usual [2]. Pregnant women are particularly vulnerable to folic acid deficiency, which can lead to the occurrence of adverse pregnancies such as preterm labor and miscarriage, as well as birth defects such as Down's syndrome, cleft lip and palate, congenital heart disease, and neural tube abnormalities [3–7]. Folic acid deficiency (red blood cell folate concentrations < 340 nmol/L or serum folate concentrations < 10 nmol/L) [8] in the body is mainly caused by inadequate intake of folic acid and genetic mutations that result in a reduced ability to metabolize folic acid [9].

*MTHFR* plays a crucial role in converting 5,10-methylenetetrahydrofolate to the active form, 5-methyltetrahydrofolate, in folate metabolism [10]. The prevalent mutation sites in *MTHFR* are 677C > T and 1298A > C. The *MTHFR*677 C > T is a mutation from C to T at the 677th site, leading to a substitution in the encoded protein from alanine to valine. Its enzymatic activity is decreased by approximately 35% in the mutant heterozygous form and by approximately 70% in the mutant pure form [11]. The *MTHFR* 1298A > C is a mutation from A to C at position 1298, which changes the encoded protein from glutamate to alanine. Enzyme activity is decreased by around 10% in the heterozygous mutated form and approximately 40% in the homozygous mutated form [12]. The *MTHFR*677 C > T and *MTHFR* 1298A > C act synergistically, and mutation at the same time will further reduce the enzyme activity [13]. Mutations in the *MTHFR* gene can impair enzyme activity, leading to compromised folate metabolism [14]. Approximately 25% of the global population are carriers of MTHFR 677 C > T, with Hispanics (47%), Europeans (36%), East Asians (30%), South Asians (12%), and Africans (9%). The frequency of MTHFR 1298A > C in Americans and Africans is 15%, and about 11% of Europeans were homozygous for the 1298A > C variant allele [15, 16]. In China, the 677TT genotype was more prevalent in the north than in the south, while the 1298CC genotype was more prevalent in the south than in the north [17]. There are ethnic and geographical differences in the distribution of *MTHFR* gene polymorphisms. Therefore, it is important to understand the distribution of *MTHFR* gene polymorphisms in this region.

In this paper, we studied the distribution of *MTHFR* gene polymorphisms among women of childbearing age (20–49 years) [18] in Tai'an and compared it with other regions to provide a scientific basis for individualized and appropriate folic acid supplementation and to reduce the incidence of adverse events among women of childbearing age in Tai'an.

## 2. Materials and Methods

### 2.1. Study Participants

A total of 243 women of childbearing age, the age ranged from 20 to 46 years, with a mean age of 31.0 ± 5.1 years, attending the eugenics clinic of Tai'an City Central Hospital between January 2018 and April 2019 were selected. The study was approved by the Ethics Committee of the Affiliated Tai'an City Central Hospital of Qingdao University (2021 Lunshen No. 50), and informed consent was obtained from all patients or their family members. Inclusion criteria are as follows: (1) > 18 years old, (2) permanent population in Tai'an area, (3) Han nationality, and (4) no history of adverse childbirth. Exclusion criteria are as follows: (1) with malignant tumor, (2) with blood system diseases, and (3) with hepatic and renal insufficiency.

### 2.2. Reagents and Instruments

Universal sequencing reaction kit, nucleic acid purification reagents, NH_4_Cl, sterilized water for injection (500 mL), TL998A Fluorescence detector (Xi'an Tianlong Science and Technology Co., Ltd.), Eppendorf high-speed centrifuge 5418, Eppendorf pipettes (10 *μ*L, 200 *μ*L, and 1000 *μ*L), centrifuge (1.5 mL), pipette tips (10 *μ*L, 200 *μ*L, and 1000 *μ*L), and EDTA anticoagulation centrifuge tubes (2 mL) were used.

### 2.3. Experimental Methods

#### 2.3.1. Specimen Collection

A volume of 1.5 mL of venous blood was collected from the patients using EDTA anticoagulation tubes. It was mixed thoroughly to prevent hemolysis or coagulation and stored at 4°C low temperatures for no longer than 24 h.

#### 2.3.2. *MTHFR* Genetic Polymorphism Detection

(1) Then, 1 mL of ammonium chloride was added to the centrifuge tube, then add 150 *μ*L of blood, and let stand for 5 min. (2) The tube was then centrifuged at 3000 rpm for 5 min, and the supernatant was discarded. (3) Then, 50 *μ*L of nucleic acid purification reagent (Beijing Sino-Era Jiyin Tech Co. Ltd.) was added and mixed. (4) Then, 1.5 *μ*L of suspension was added to the corresponding universal kit (Beijing Sino-Era Jiyin Tech Co. Ltd.) for sequencing reaction. The pipette tip was checked for any liquid residue on its front, and the cap was tightly fastened. The tube was inverted several times to ensure thorough mixing, and the wall of the tube was flicked to remove bubbles from the liquid surface. A microcentrifuge was used briefly to remove droplets attached to the tube's wall and using the software (V1.0.30/V2.0.714/V2.2.010) to get a mixture of numbered. (5) The TL998A fluorescence detector was used for testing. (6) The fluorescence profile images were reviewed for genotyping.

### 2.4. Statistical Analysis

We applied the Hardy–Weinberg law of genetic equilibrium to test the population representativeness of the samples. SPSS 25.0 was used for statistical analyzed. The measurement data were presented as mean ± SD ($\bar{X}\pm s$), the counting data were represented by examples (%), compared between groups using the chi-square (*χ*^2^) test, and hierarchical cluster analysis was used for comparison of mutation rate. *p* < 0.05 indicates that the difference is statistical significance.

## 3. Results

### 3.1. Hardy–Weinberg Genetic Equilibrium and Genotype Distribution

The Hardy–Weinberg genetic equilibrium test for *MTHFR* 677C > T and 1298A > C polymorphisms in 243 women of childbearing age showed that the results were all *p* > 0.05, which were consistent with Hardy–Weinberg's law of genetic equilibrium, indicating that the selected samples were representative of the population.

**Figure 1:**
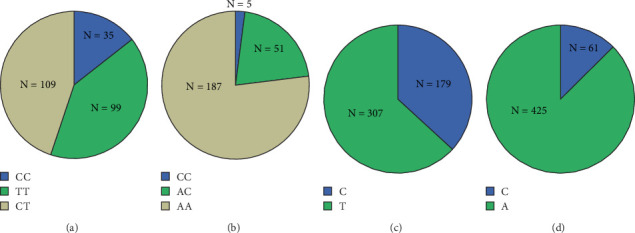
The proportion of different genotypes of *MTHFR*. (a) Distribution of *MTHFR* 677C > T genotypes in fertile females in Tai'an. (b) Distribution of *MTHFR* 1298A > C genotypes in fertile females in Tai'an. (c) Distribution of *MTHFR 677*C > T alleles in fertile females in Tai'an. (d) Distribution of *MTHFR* 1298A > C alleles in fertile females in Tai'an.

The distribution of *MTHFR* 677C > T genotypes included 35 wild pure (CC) cases with a genotype frequency of 14.4%, 109 mutant heterozygous (CT) cases with a genotype frequency of 44.9%, and 99 mutant pure (TT) cases with a genotype frequency of 40.7% (Figure 1a). The distribution of *MTHFR* 1298A > C genotypes included 187 wild pure (AA) cases with a genotype frequency of 76.9%, 51 mutant heterozygous (AC) cases with a genotype frequency of 21.0%, and 5 mutant pure (CC) cases with a genotype frequency of 2.1% (Figure 1b). The allele distribution of *MTHFR* 677C > T included 179 cases of allele C with a frequency of 36.8% and 307 cases of allele T with a frequency of 63.2% (Figure 1c). The allele distribution of *MTHFR* 1298A > C included 61 cases of allele C with a frequency of 12.6% and 425 cases of allele A with a frequency of 87.4% (Figure 1d).

### 3.2. The Linkage Distribution of *MTHFR 677C > T* and 1298A > C Genes

The CC/AA genotype accounted for 16 cases (6.6% of the total), the CC/AC genotype for 14 cases (5.8% of the total), the CC/CC genotype for 5 cases (2.0% of the total), the CT/AA genotype for 74 cases (30.5% of the total), the CT/AC genotype for 35 cases (14.4% of the total), the TT/AA genotype for 97 cases (39.9% of the total), the TT/AC genotype for 2 cases (0.8% of the total), and the TT/CC and CT/CC genotypes for 0 cases. The TT/AC genotype was found in two cases, accounting for 0.8% of the total, and the TT/CC and CT/CC genotypes were both zero cases (Table 1).

### 3.3. Comparison of *MTHFR* 677C > T and 1298A > C Gene Polymorphisms in Different Regions

The differences in *MTHFR* 677C > T gene polymorphism in women of childbearing age in Tai'an were not statistically significant compared with those in Haiyang (TT, 36.5%), Zibo (TT, 43.6%), and Zhengzhou (TT, 38.3%) (*p* > 0.05) and statistically significant compared with those in Changchun (TT, 31.7%), Chifeng (TT, 32.0%), Xinjiang (TT, 25.1%), Taiyuan (TT, 29.3%), Wuhan (TT, 15.8%), Lishui (TT, 14.0%), Shenzhen (TT, 15.8%), Yunnan (TT, 14.1%), and Qionghai (TT, 6.1%) (*p* < 0.05). The frequencies of 677T allele and 677 TT genotype were higher in Zibo (65.6% and 43.6%, respectively), Tai'an (63.2% and 40.7%, respectively) and low in Shenzhen (29.3% and 15.8%, respectively) and Qionghai (22.1% and 6.1%, respectively), which was consistent with the trend of high in the north and low in the south (Table 2).

### 3.4. Comparison of *MTHFR* 1298A > C Gene Polymorphisms in Different Regions

The differences in *MTHFR* 1298A > C gene polymorphism in women of childbearing age in Tai'an were not statistically significant compared with Haiyang (CC, 1.1%), Zibo (CC, 1.4%), Changchun (CC, 2.2%), Chifeng (CC, 1.9%), Xinjiang (CC, 3.6%), and Zhengzhou (CC, 2.0%) and statistically significant compared with Taiyuan (CC, 2.0%), Wuhan (CC, 3.6%), Lishui (CC, 2.9%), Shenzhen (CC, 5.8%), Yunnan (CC, 2.7%), and Qionghai (CC, 7.1%) (*p* < 0.05). The 1298C allele and the 1298CC genotype frequencies were low in Zibo (11.2% and 1.4%, respectively) and Taian (12.6% and 2.1%, respectively) and higher in Shenzhen (22.4% and 5.8%, respectively) and Qionghai (24.9% and 4.1%, respectively), which was consistent with the trend of high in the north and low in the south. The 1298C allele and the 1298CC genotype frequencies were significantly higher in the southern than in the northern populations (Table 3).

### 3.5. Mutation Rates of *MTHFR* 677C > T and 1298A > C Genes in Different Regions

**Figure 2:**
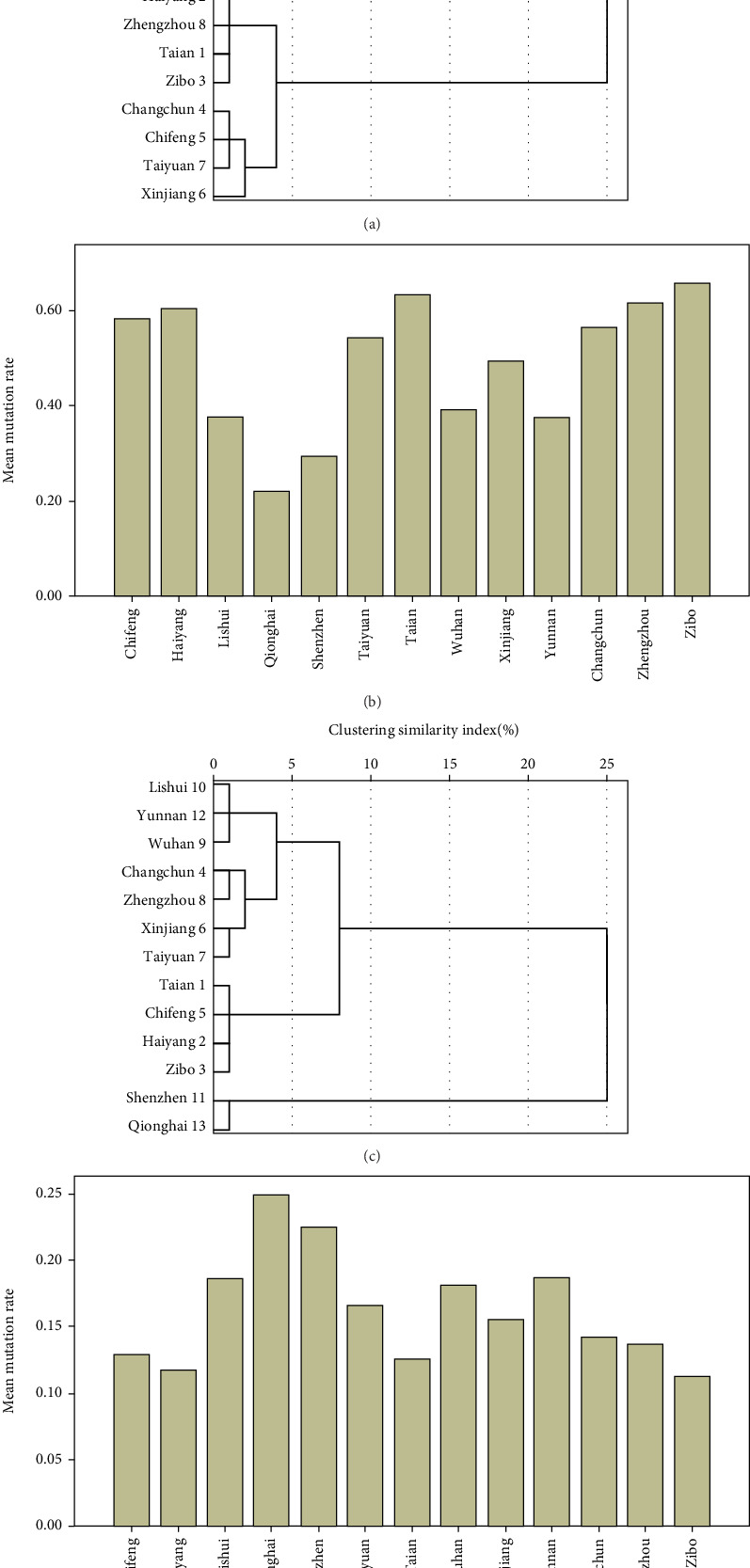
*MTHFR* gene polymorphisms in different regions. (a) Tree of *MTHFR* 677C > T mutation rate in different regions. (b) Bar graph of *MTHFR* 677C > T mutation rate in different regions. (c) Tree of *MTHFR* 1298A > C mutation rate in different regions. (d) Bar graph of *MTHFR* 1298A > C mutation rate in different regions.

By performing a hierarchical cluster analysis on the mutation rate of *MTHFR* 677C > T gene, the results showed that Lishui, Yunnan, and Wuhan were the first clusters; Shenzhen and Qionghai were the second category; Haiyang, Zhengzhou, Tai'an, Zibo, and Changchun were the third category; and Chifeng, Taiyuan, and Xinjiang were the fourth category. The results showed that the mutation rate of *MTHFR* 677C > T gene had obvious geographical characteristics (Figure 2a,b). By stratified cluster analysis of the mutation rate of *MTHFR* 1298A > C gene, the results showed that Lishui, Yunnan, and Wuhan were the first category; Changchun, Zhengzhou, Xinjiang, and Taiyuan were the second category; Tai'an, Chifeng, Haiyang, and Zibo were the third category; and Shenzhen and Qionghai were the fourth category. The results showed that the geographical characteristics of the mutation rate of *MTHFR* 1298A > C gene were not obvious (Figure 2c,d).

## 4. Discussion

The results of this study showed that the *MTHFR* 677C > T mutation was predominantly mutant heterozygous, and the *MTHFR* 1298A > C was predominantly wild heterozygous in women of childbearing age in the Tai'an area. Approximately 93.4% of women of childbearing age have abnormal *MTHFR* enzyme activity, and approximately 87.6% of women of childbearing age have moderate to high risks of folate utilization. The *MTHFR* 677C > T gene had obvious geographical characteristics.

The gene encoding the *MTHFR* enzyme was situated at the distal end of chromosome 1's short arm (1p36.3) and primarily codes a dimeric protein [19]. MTHFR plays a very important role in folate metabolism and has a critical role in epigenetics such as DNA methylation. There was growing evidence suggesting that DNA methylation in healthy populations and individuals with various diseases may be associated with *MTHFR* [20]. The primary function of *MTHFR* was to convert 5,10-methylenetetrahydrofolate into biologically active 5-methyltetrahydrofolate. 5-methyltetrahydrofolate can further enter the methyl transfer pathway and indirectly provide methyl for DNA methylation and protein methylation by facilitating homocysteine remethylation, thereby maintaining low homocysteine levels in the bloodstream. However, folic acid supplementation was not as good as it gets, and oversupplementation can lead to a range of health risks [21–24]. Therefore, folic acid supplementation needs to be individualized.

Our study results showed that in women of reproductive age in the Tai'an area, the frequencies of the *MTHFR* 677C > T CC, CT, and TT genotypes were 14.4%, 44.9%, and 40.7%, respectively. The allele frequencies of C and T distributions were 36.8% and 63.2%, with a relatively high rate of allele mutations. Previous studies showed that the frequency of the T allele in the Shandong population was about 63% [25], which was consistent with the results of this study. The frequencies of *MTHFR* 1298A > C AA, AC, and CC genotypes were 77.0%, 21.0%, and 2.0%, respectively. The allele A and C distribution frequencies of 87.4% and 12.6%, respectively. A study of 10138 women in Shandong showed that the frequency of C allele distribution was about 13.2% [26], which was basically consistent with the results of our study.

Genetic polymorphisms in folate metabolizing enzymes determine an individual's ability to utilize folate, and *MTHFR* enzyme activity can be classified into four classes based on the *MTHFR* 677C > T and *MTHFR* 1298A > C chain genotypes: normal enzyme activity (CC/AA), mild metabolic impairment (CC/AC), moderate metabolic impairment (CC/CC and CT/AA), and severe metabolic impairment (CT/AC, CT/CC, TT/AA, TT/AC, and TT/CC) [27, 28]. The results of this study showed that the proportion of women at high risk of folic acid use in Tai'an reached 55.1%, and more than half of the women of childbearing age in this region had severe folic acid metabolism disorders. The proportion of women with moderate or severe risk of folic acid use reached 87.6%. The results of this study are significantly higher than those of previous studies [29, 30]. This study showed that the mutation rate of *MTHFR* 677C > T gene in Tai'an exhibited significant geographical characteristics, while the mutation rate of *MTHFR* 1298A > C gene did not show such distinct geographical patterns. These differences may be related to the small sample size and study region of our study. Women of childbearing age in this region should increase the dose and duration of folic acid and dietary folic acid supplementation to reduce the risk of neonatal birth defects and maternal diseases.

In conclusion, the *MTHFR* 677C > T and 1298A > C gene polymorphisms were identified in women of childbearing age in Tai'an area, and the risk ratio of medium and high folic acid utilization in women of childbearing age were higher. It suggests that it is necessary to detect the folic acid gene polymorphism in women of childbearing age in Tai'an. Folic acid supplementation doses were individualized to reduce the incidence of adverse events.

## Figures and Tables

**Table 1:** Distribution of *MTHFR*677C > T/1298A > C genotypes among women of childbearing age in Tai'an (%).

***MTHFR677C > T***	***MTHFR1298A > C***		
	**AA**	**AC**	**CC**
CC	16 (6.6%)	14 (5.8%)	5 (2.0%)
CT	74 (30.5%)	35 (14.4%)	0 (0.0%)
TT	97 (39.9%)	2 (0.8%)	0 (0.0%)

**Table 2:** Comparison of *MTHFR* 677C > T gene polymorphisms in different regions.

**Region**	***MTHFR 677C > T***			**χ** ^2^	**p**
	**CC**	**CT**	**TT**		
Tai'an	35	109	99		
Haiyang	58	177	135	1.131	0.568
Zibo	130	457	454	0.980	0.613
Changchun	648	1676	1081	9.149	0.010
Chifeng	101	339	207	6.094	0.047
Xinjiang	111	202	105	22.816	≤ 0.001
Taiyuan	219	538	313	13.276	≤ 0.001
Zhengzhou	399	1236	1017	0.538	0.764
Wuhan	1595	1953	668	117.398	≤ 0.001
Lishui	253	312	92	92.230	≤ 0.001
Shenzhen	1406	670	390	175.707	≤ 0.001
Yunnan	116	139	42	65.376	≤0.001
Qionghai	756	390	75	298.714	≤0.001

**Table 3:** Comparison of *MTHFR* 1298A > C gene polymorphisms in different regions.

**Region**	***MTHFR 1298A > C***			**χ** ^2^	**p**
	**AA**	**AC**	**CC**		
Tai'an	187	51	5		
Haiyang	287	79	4	0.969	0.616
Zibo	822	204	15	0.774	0.679
Changchun	2511	818	76	1.225	0.542
Chifeng	492	143	12	0.158	0.924
Xinjiang	303	100	15	2.184	0.336
Taiyuan	735	314	21	6.911	0.032
Zhengzhou	1977	623	52	0.783	0.676
Wuhan	2836	1230	150	10.028	0.007
Lishui	431	207	19	10.640	0.005
Shenzhen	1504	819	143	24.992	≤ 0.001
Yunnan	194	95	8	8.769	0.012
Qionghai	699	435	87	34.370	≤ 0.001

## Data Availability

All data and materials were truly available.
